# Anatomical cross-sectional area of the quadriceps femoris and sit-to-stand test score in middle-aged and elderly population: development of a predictive equation

**DOI:** 10.1186/s40101-016-0099-1

**Published:** 2016-06-29

**Authors:** Akira Saito, Ryoichi Ema, Takayuki Inami, Sumiaki Maeo, Shun Otsuka, Mitsuru Higuchi, Shigenobu Shibata, Yasuo Kawakami

**Affiliations:** Faculty of Sport Sciences, Waseda University, 2-579-15 Mikajima, Tokorozawa, Saitama Japan; Graduate School of Engineering and Science, Shibaura Institute of Technology, 307 Fukasaku, Minuma-ku, Saitama Japan; Research Fellow of Japan Society for the Promotion of Science, 5-3-1 Kojimachi, Chiyoda-ku, Tokyo Japan; Graduate School of Sport Sciences, Waseda University, 2-579-15 Mikajima, Tokorozawa, Saitama Japan; Institute of Advanced Active Aging Research, 2-579-15 Mikajima, Tokorozawa, Saitama Japan; School of Advanced Science and Engineering, Waseda University, 2-2 Wakamatsu, Shinjuku-ku, Tokyo Japan

**Keywords:** Magnetic resonance imaging, Muscle size, Aging, Trunk motion, Multiple regression analysis

## Abstract

**Background:**

Although the sit-to-stand (STS) test score has been shown to relate to the strength and size of the quadriceps femoris (QF) for elderly population, it is unknown whether this relationship is influenced by a posture (i.e., the trunk being allowed to stoop or not) during the STS test. The present study investigated the relationship between STS test score and QF anatomical cross-sectional area (ACSA) in the middle-aged and elderly population with regard to the difference in the posture during STS test, and aimed to develop an accurate predicting equation of the QF ACSA from the STS test score.

**Methods:**

105 males (40–81 years) and 113 females (41–79 years) participated in the present study, then the subjects were divided at random as validation and cross-validation groups. Mid-thigh QF ACSA was determined by magnetic resonance imaging. Subjects performed a 10-repeated STS as fast as possible in two conditions: (1) with the trunk being allowed to stoop during the sitting phases, and (2) kept upright throughout the test. A power index of the STS test score was calculated based on an equation obtained in a previous study using the time taken for each test condition, the thigh and shank lengths, and body mass. In the validation group (*n* = 109), a stepwise multiple linear regression analysis was performed to create a predictive model of the ACSA with sex, age, the STS time, and power for both conditions as independent variables. The formulated predictive equation was examined in the cross-validation group (*n* = 109).

**Results:**

In the validation group, a stepwise regression analysis revealed that STS power with upright trunk condition, sex, and age but not with the stooping condition, were selected as variables to predict QF ACSA (*R*^2^ = 0.64, *P* < 0.001). There was no systematic error for the relationship between predicted and measured values in the cross-validation group.

**Conclusions:**

These results indicate that STS test score with upright trunk condition is one of the indices of QF muscle size of the middle-aged and elderly population. The estimated predicting equation should be useful in clinical and practical settings for the health promotion.

## Background

It is well known that strength of skeletal muscle decreases with aging [1, 2], prominently in the quadriceps femoris (QF) [3, 4]. The decline in knee extension torque in the elderly population can compromise the capacity to perform daily physical activities such as walking [5] or standing up from a chair [6], and increase the risk of falls [7, 8], thereby reducing the ability to keep physical independency and the quality of life. Such age-related reduction in skeletal muscle strength is associated with the loss of skeletal muscle mass [2]. Therefore, assessment of QF muscle size, especially for the elderly population, has been a key topic in the field of exercise physiology and health sciences.

The QF muscle size can be accurately measured with the magnetic resonance (MR) imaging as the anatomical cross-sectional area (ACSA) [9–11]. However, the MR system is not easily accessible and thus not suitable for large-sample assessments. With this background, QF ACSA has been assessed from the muscle strength or power of individuals [1, 12–15]. From those studies, the ability to stand up from a chair assessed by a sit-to-stand (STS) test has been proposed to be one of the useful indices of skeletal muscle strength [12, 16–21]. The advantage of the STS test is easiness and safeness of the measurement for the elderly population, and the STS test score can be used as muscle power index which is closely correlated with maximal power of the lower limb [21] and QF ACSA [12].

When we stand up from a chair, the exerted force and muscle activation of QF are greater than those of other muscle groups in the lower limb [22]. Following lift-off from a chair, a trunk-raising movement accelerates elevation of the center of gravity [23]. However, this movement is provided not only by QF but also by hip extensor muscles. Hence, the trunk position during the STS being upright or stooped forward during the sitting phase would influence the muscle activity and force of QF. This notion also comes from the fact that the estimated QF force is greater during single leg squat with the trunk kept upright than being stooped [24]. It is therefore likely that the STS test score can be a better index to estimate the QF ACSA, when measured with the trunk being kept upright throughout the test than stooped during the sitting phase. Although the peak vertical acceleration of center of mass during STS in the young was shown to be not significantly different between normal and trunk stooped conditions [25], no studies have examined whether the difference in the posture during the STS influences its test score for the elderly. In fact, the QF muscle activity during a body mass-based squat movement is approximately four-fold higher in the elderly than the young [26]. To clarify this would provide important methodological and practical information regarding how the test should be performed.

The purpose of the present study was to investigate the relationship between STS test score and QF ACSA in the middle-aged and elderly population with respect to the difference in the posture during the STS test, and to develop an accurate predicting equation of QF ACSA from the STS test score. The STS test was performed with the two different posture conditions: the trunk stooped during the sitting phases or kept upright throughout the test. We hypothesized that STS test score with the upright trunk condition would be a suitable independent variable to estimate QF ACSA.

## Methods

### Subjects

Japanese 105 males (40–81 years) and 113 females (41–79 years) participated. The age distribution of the subjects for each decade was as follows: forties, 10 males and 11 females; fifties, 7 males and 12 females; sixties, 47 males and 56 females; seventies, 40 males and 34 females; eighties, 1 male. All subjects were medically screened before the measurements. They were healthy and free from cardiovascular, metabolic, and immunologic disorders, as well as orthopedic abnormality. To calculate a predictive equation for the QF ACSA, the subjects were divided at random as the validation and the cross-validation groups (Table 1). The present study was approved by the Ethical Committee on Human Research of Waseda University (2014-G003). Each subject was informed of the purpose, procedures, and possible risks of the measurements of the present study. The protocols proceeded in accordance with the guidelines in the Declaration of Helsinki.

**Table 1:** Physical characteristics of subjects in validation and cross-validation groups

	Males		Females	
	Validation group (*n* = 53)	Cross-validation group (*n* = 52)	Validation group (*n* = 56)	Cross-validation group (*n* = 57)
Age (year)	65.7 ± 9.0	66.5 ± 8.5	64.0 ± 8.4	64.1 ± 9.3
Height (cm)	166.7 ± 6.1	168.9 ± 6.8	154.3 ± 5.0	155.5 ± 5.6
Body mass (kg)	65.1 ± 8.6	68.2 ± 10.7	54.5 ± 8.8	53.4 ± 6.8
Body mass index (kg/m^2^)	23.4 ± 2.6	23.8 ± 2.8	22.8 ± 3.4	22.0 ± 2.5

Values are mean and standard deviation

### Measurement of cross-sectional area in the quadriceps femoris

A series of cross-sectional images of the right thigh was scanned using MR scanner with a body coil (Signa EXCITE 1.5T; GE Medical Systems, USA). T1-weighted spin-echo transaxial images were collected with the following parameters: echo time, 10 ms; repetition time, 520 ms; slice thickness, 10 mm; gap, 0 mm; matrix, 256 × 192; field of view, 240 mm. Subjects lay supine with their arms and legs fully extended and relaxed in the magnet bore. Scanned MR images were transferred to a computer, and QF ACSA was measured by manually tracing the outline of muscle tissue using software (ImageJ, MIPAV; National Institutes of Health, USA). We took care of excluding visible adipose and connective tissue from individual ACSAs. We selected an image of mid-thigh according to a marker attached at the middle between the great trochanter and lateral condyle of femur, because the peak ACSA of the QF was located at the mid-thigh [27, 28]. The QF ACSA was denoted absolute (cm^2^) and adjusted values which were divided by two thirds power of body mass (cm^2^/mass^2/3^) [10].

### Sit-to-stand test

**Fig. 1:**
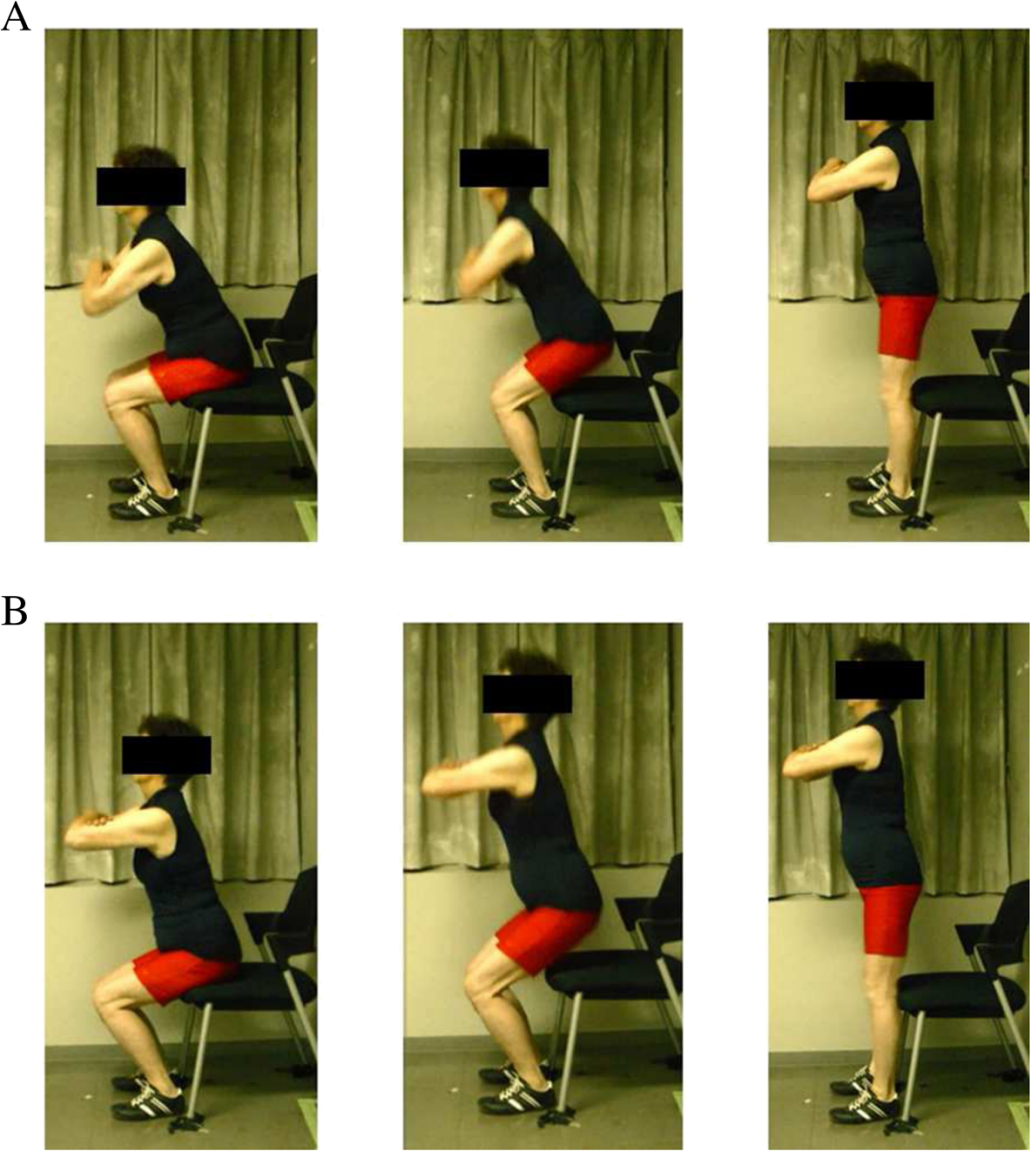
Representative motions during sit-to-stand test. Subjects performed the sit-to-stand test **a** with stooping during the sitting phase and **b** with the trunk kept upright

The STS test was defined as rising from a chair with two different postures, with the trunk being allowed to stoop during the sitting phase (Fig. 1a) or kept upright throughout the test (Fig. 1b). The STS test began with subjects standing in front of a chair (40 cm height) and feet on a flat floor placed about shoulder width apart. The subjects were asked to sit down from a standing position then to stand up 10 times as fast as possible. They were instructed to touch their buttocks on a seat of the chair in a sitting position and to stand up fully between individual repetitions. Prior to the test, familiarization trials were performed for positioning and learning of each movement. The time was measured using a stopwatch. The test started when the supervisor said, after counting down from three, “go” and finished when the participants fully stood up on the 10th repetition.

A power index of the STS test score was calculated by the following equation [12]:

1 $$ \mathrm{Power}=\frac{\left(L-0.4\right)\times \mathrm{body}\ \mathrm{mass}\times \mathrm{g}\times 10}{T} $$

where 0.4 (m), *L* (m), g (m/s^2^), and *T* (s) indicate height of the chair, the thigh and shank lengths (distance between the great trochanter of femur and malleolus lateralis), acceleration of gravity (9.8 m/s^2^), and the time of STS test score, respectively. This equation can calculate the average mechanical power during the STS test using the variables of individual body size and the time of STS test. For instance, the distance of the center of gravity during the STS movement is expressed as the difference between the leg length and height of the chair. Then, the mechanical work during the STS test is calculated by the distance of the center of gravity, body mass, acceleration of gravity, and number of repetitions.

### Measurement of isometric knee extension torque during maximal voluntary contraction

Subjects performed an isometric knee extension of the right side during maximal voluntary contraction (MVC) on a specially designed dynamometer (VTK-002, Vine, Japan). They seated on the device with the hip and knee joints fixed at 80° and 70° (anatomical position = 0°), respectively. Prior to the measurement, the subjects performed adequate warm-up, consisting of submaximal contractions of 30, 50, and 80 % of maximal effort to familiarize themselves with the measurement. After the rest period of 1 min, the subjects attempted two MVCs. Each MVC was approximately 3 s and subjects rested for 1 min between MVC attempts. If the generated peak torque differed by more than 10 % between the attempts, an additional attempt was imposed. The torque signals were amplified by a strain amplifier (DPM-711B, Kyowa, Japan) and AD converted (Power Lab, ADInstruments, Australia) into a computer at 1000 Hz with a low-pass filter (cut-off frequency, 10 Hz). The highest knee extension torque during MVC was adopted. The intraclass correlation coefficient between two measurements was 0.989. The knee extension toque was expressed both as absolute value (Nm) and ratio of the torque to QF ACSA (Nm/cm^2^) [29].

### Measurement of leg extension power

Power of the lower limb was assessed using a multi-joint leg extension apparatus (Anaeropress 3500, Combi, Japan). The load of leg extension was set according to the individuals’ body mass. Subjects sat back on the devise, positioned feet on the sliding foot plate with knee joint of 90°, and hip was securely strapped to the seat. After several warm-up trials, leg extension was performed five times with 15-s rest intervals between the attempts. They were asked to extend their leg as fast as possible. The highest leg extension power was adopted. The intraclass correlation coefficient between the highest two values was 0.980. The leg extension power was also expressed as absolute value (W) and ratio of the power to QF ACSA (W/cm^2^).

### Statistics

The difference of absolute and adjusted values of body mass index and QF ACSA, knee extension torque, and leg extension power between males and females was compared using unpaired Student’s *t* test. Time and power of STS test score were analyzed using a two-way (posture × sex) analysis of variance with repeated measures. Correlation coefficients between QF ACSA and each of the STS time, STS power, knee extension torque, and leg extension power were examined using a Pearson’s correlation analysis. The differences of physical characteristics between the validation and cross-validation groups were compared using unpaired Student’s *t* test. A stepwise multiple linear regression analysis was performed to create a predictive model of absolute QF ACSA value for the validation group. Sex (males, 0; females, 1), age, and the time and power of STS test score at each posture condition were entered into the stepwise regression as independent variables if they represented a significant contribution to the explained variance (*F* to enter ≤0.05, *F* to remove ≥0.10). In the cross-validation group, the difference of the ACSA between measurement and estimated values was compared by paired Student’s *t* test. A Bland-Altman plot was constructed to determine if there was a systematic error between the measured and estimated values [30]. The level of significance was set at *P* < 0.05. Statistical analyses were performed using the IBM SPSS Statistics software (version 22.0; IBM, Japan).

## Results

### Muscle size, muscle strength, and sit-to-stand test score

**Fig. 2:**
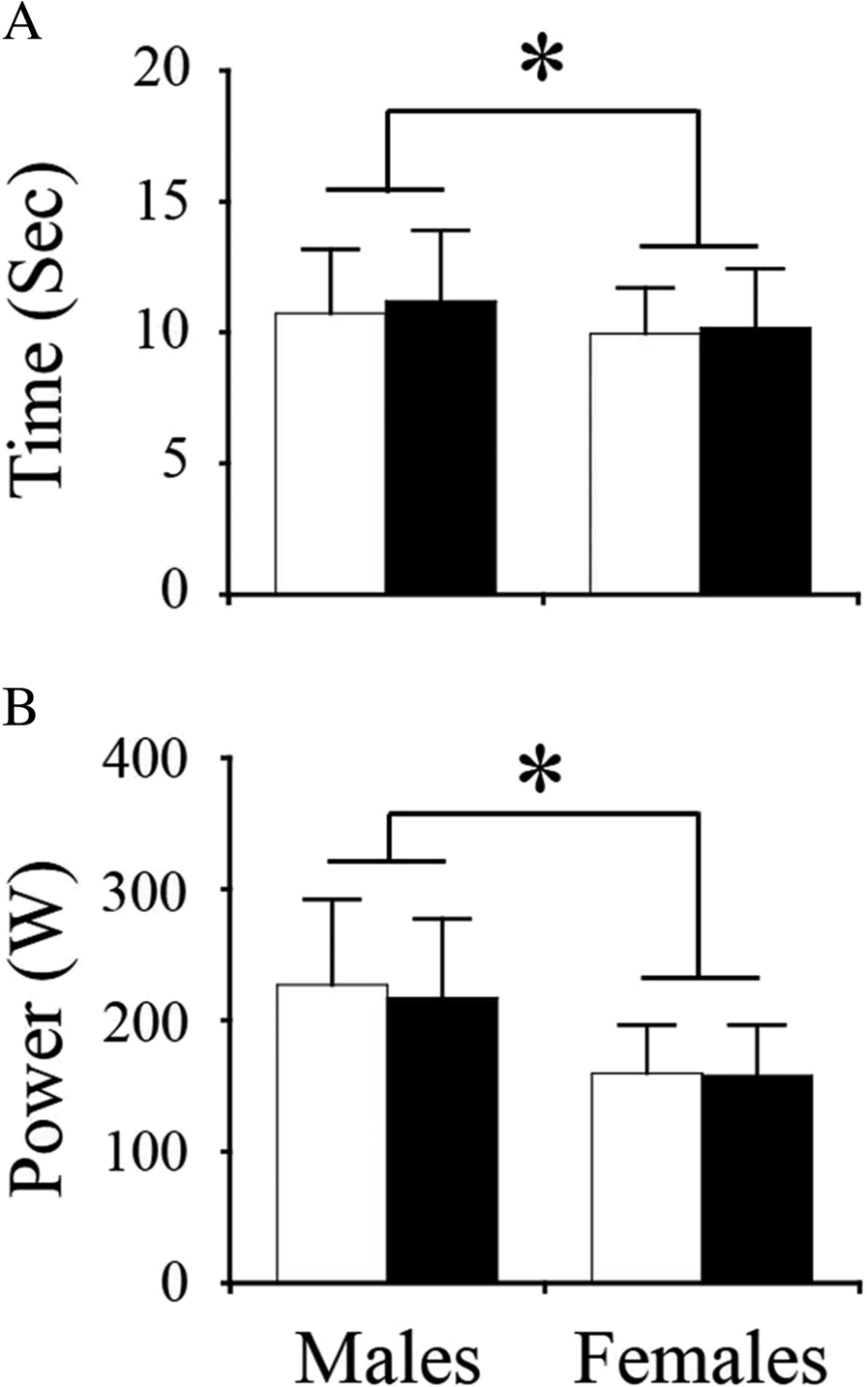
Sit-to-stand test scores of the two different conditions. The time taken for sit-to-stand test (**a**) and the power index of sit-to-stand test score (**b**). *White* and *black bars* indicate the trunk stooping and the trunk kept in the upright condition, respectively. *P < 0.05

Absolute and adjusted values of QF ACSA, knee extension torque and leg extension power of males were significantly greater than those of females (Table 2). The time taken for STS test score of males was significantly longer than that of females regardless of the test conditions (Fig. 2a). The power of STS test score of males was significantly greater than that of females regardless of the test conditions (Fig. 2b). No significant interaction or the main effect of the posture from the STS test scores was observed in both variables.

**Table 2:** Muscle size and physical performances in elderly males and females

	Males	Females
ACSA (cm^2^)	47.9 ± 9.6***	33.7 ± 7.9
ACSA (cm^2^/mass^2/3^)	2.9 ± 0.5***	2.3 ± 0.4
Torque (Nm)	170.0 ± 51.6***	106.0 ± 30.5
Torque (Nm/cm^2^)	3.5 ± 0.9**	3.2 ± 0.9
Leg extension power (W)	1090.6 ± 390.9***	646.4 ± 206.4
Leg extension power (W/cm^2^)	22.7 ± 6.4***	19.5 ± 6.1

Values are mean and standard deviation

*ACSA* anatomical cross-sectional area

**P < 0.01; ***P < 0.001 vs. females

**Fig. 3:**
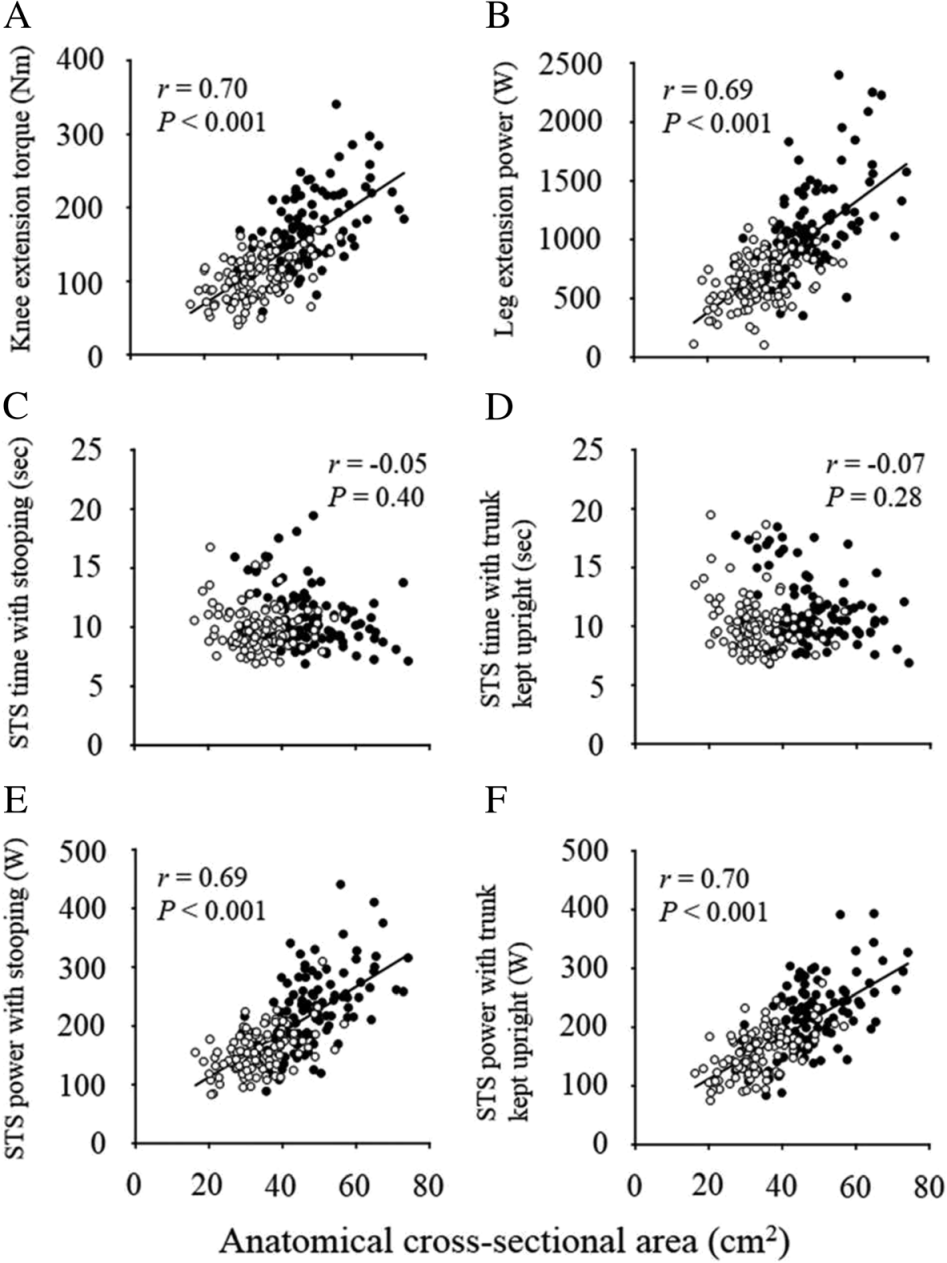
The relationship between quadriceps femoris muscle size and performance of physical function. Correlation coefficients between anatomical cross-sectional area of quadriceps femoris and **a** knee extension torque, **b** leg extension power, **c**, **d** the time, and **e**, **f** the power of sit-stand test score for each condition were presented. *Close* and *open circles* indicate males and females, respectively

In pooled data of males and females, a significant correlation was observed between QF ACSA and knee extension torque, leg extension power, power of STS test score with the trunk stooped and upright conditions (Fig. 3). The ACSA was not significantly correlated to the time of STS test score with stooping and the upright trunk conditions.

### Validation of predictive equation of cross-sectional area of quadriceps femoris

**Fig. 4:**
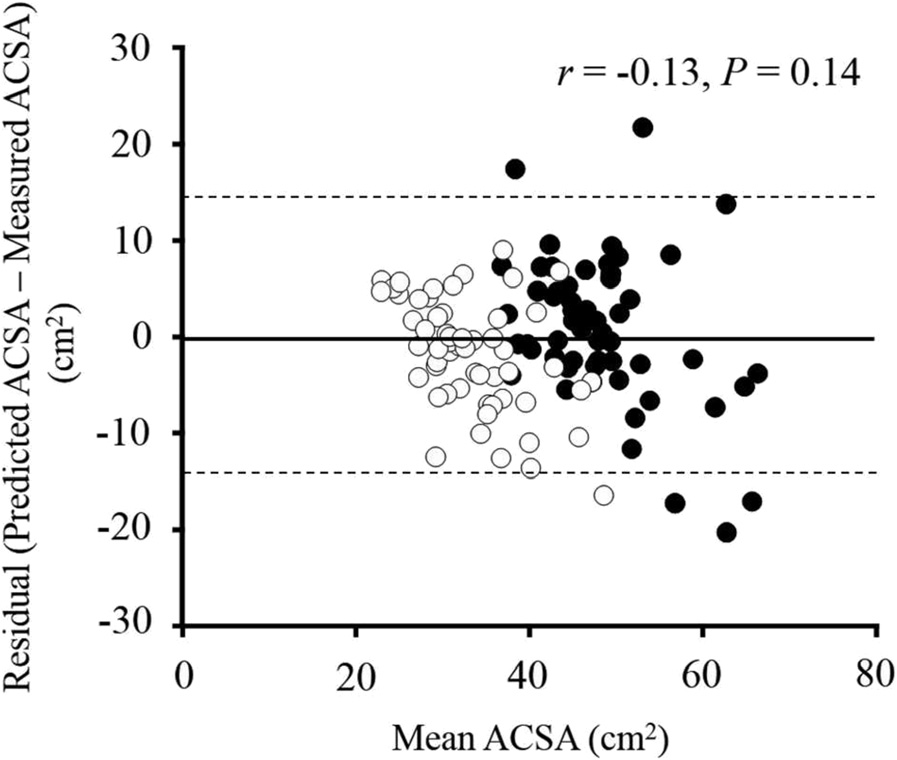
The relationship between residuals and mean of the predicted and measured values in the cross-validation group. A *solid line* is mean of residuals and two *dashed lines* are two standard deviations of the residual values. *Close* and *open circles* indicate males and females, respectively. The residual value is the difference between predicted and measured anatomical cross-sectional area (ACSA) in the quadriceps femoris

No significant difference in physical characteristics between the validation and cross-validation groups was observed for both males and females (Table 1). The stepwise multiple linear regression revealed that the power of STS test score with trunk kept in the upright condition was a significant predictor of the QF ACSA (*R*^2^ = 0.44, *P* < 0.001) (Table 3, step 1). The addition of the sex (*R*^2^ = 0.59, *P* < 0.001) and age (*R*^2^ = 0.64, *P* < 0.001) improved the strength of the estimate (Table 3, steps 2 and 3). No significant difference between measured and estimated QF ACSA was observed in the cross-validation group. The Bland-Altman plot showed no significant systematic error between the residuals and the mean of predicted and measured QF ACSA in the cross-validation group (Fig. 4).

**Table 3:** Stepwise multiple linear regression analysis predicting ACSA of quadriceps femoris

Independent variables	Multiple regression equation	*R*	*R* ^2^	*P*
Step 1				
*X*_1_: STS power with kept upright	ACSA = 13.41 + 0.14*X*_1_	0.67	0.44	<0.001
Step 2				
*X*_1_: STS power with kept upright	ACSA = 25.86 + 0.10*X*_1_ − 9.68*X*_2_	0.76	0.59	<0.001
*X*_2_: sex (males, 0; females, 1)				
Step 3				
*X*_1_: STS power with kept upright	ACSA = 54.55 + 0.07*X*_1_ − 11.66*X*_2_ − 0.343*X*_3_	0.80	0.64	<0.001
*X*_2_: sex				
*X*_3_: age				

*ACSA* anatomical cross-sectional area, *STS* sit-to-stand

## Discussion

The present study investigated the relationship between STS test score and QF ACSA in the middle-aged and elderly population, with the aim of developing an accurate predicting equation of QF ACSA from STS test score. The main findings of the present study were that a stepwise multiple linear regression analysis showed that the power of STS test score with the upright trunk condition, sex, and age were selected as an independent variable to predict the QF ACSA. These results supported our hypothesis that the STS test score with the upright trunk condition would be a better independent variable to estimate QF ACSA.

ACSA can reflect force-generation capacity of skeletal muscles of young [13, 31–33] and elderly populations [13, 14, 32]. The previous studies have reported that correlation coefficients between the QF ACSA and knee extension torque in the elderly population ranged from 0.48 to 0.85 [12–14]. The results of the present study indicated that QF ACSA was significantly correlated to knee extension torque (*r* = 0.70) and leg extension power (*r* = 0.69) (Fig. 3). These findings support the previous reports [12–14] and indicate that correlations between the QF ACSA and maximal knee extension torque are moderate or high.

In the validation group, a stepwise linear regression analysis extracted the power of STS test score with the upright trunk condition, sex and age as significant predictors of the QF ACSA. It could account for 64 % of variance in the ACSA (*R*^2^ = 0.64) (Table 3). In the cross-validation group, a Bland-Altman plot showed that no systematic error was found between residuals and mean of the developed predictive equation of QF ACSA (Fig. 4). These results suggest that the predictive equation of the present study can adequately estimate the QF ACSA for a wide range of the middle-aged and elderly population by the power of STS test score with the trunk kept upright, sex, and age.

In the predictive equation in step 1, the power of STS test score with the trunk upright condition was selected as a significant predictor of the QF ACSA (Table 3). Kanehisa and Fukunaga [18] showed that an age-related decline in power of STS test score was parallel to the decline in knee extension torque in 556 females aged 50 to 94 years. They suggested that power of STS test score can be an effective measurement representing the age-related change in the QF capacity. Since the power of STS test score is closely correlated to knee extension torque during MVC [12], the power of STS test score would be primarily selected by the multiple regression analysis for estimating the QF ACSA.

It has been reported that although the age-related reduction in the knee extension strength was similar between males and females, the age-related loss of the power of STS test score in females was greater than that in males [20], suggesting that a greater impairment in power generation capability of females than males during the quick or explosive movement. This is also supported by the current results that ratio of knee extension torque and leg extension power to the QF ACSA of males was significantly higher than that of females (Table 2). Thus, the sex difference of age-related change in the control strategies during dynamic movements, such as a STS task, may be one of the potential factors selecting sex and age as a significant predictor of the QF ACSA.

QF ACSA was significantly correlated to the power of STS test score with the trunk stooped (*r* = 0.69) and with the upright conditions (*r* = 0.70) (Fig. 3). Lindemann et al. [15] assessed the power during standing from a chair for 88 elderly females using a linear encoder and showed a significant relationship between power during standing task and a total muscle ACSA of the mid-thigh (*r* = 0.51). This correlation coefficient was lower than that of the present result. The present study calculated the power index from three variables in individual subjects such as body mass, leg length, and time taken for STS test, which strongly correlates to the QF ACSA (Fig. 3). Moreover, we determined muscle ACSA from the QF, while Lindemann et al. [15] used a total muscle ACSA of the mid-thigh (including other muscles such as hamstrings). The STS test score of elderly males and females is strongly correlated to the muscle strength of the QF than that of the hamstrings [34]. Thereby, the power determination during STS test and the target of muscle ACSA may determine the relationship between the power during STS test score and muscle size indices for middle-aged and elderly population.

As mentioned earlier, a trunk raising movement during standing task from a chair accelerates elevation of the center of gravity in the initial phase [23], and other studies have reported that the trunk position during squat exercises influences muscle activity of the QF [24, 35]. However, Fujimoto and Chou [25] compared the peak vertical acceleration of center of mass during normal and trunk stooped STS tasks of the young, and showed that no significant difference was observed between both tasks. The present result also indicated that a similar value was found in both of the time and power of STS test scores between the stooped and upright trunk conditions (Fig. 2). These discrepancies may be attributed to the differences in the protocols under which the tests were performed. The previous studies which showed that trunk position influenced muscle activity of QF [24, 35] performed the test with a fixed pace [24] or carrying one-repetition maximum load [35], while the present study and the study of Fujimoto and Chou [25] performed the test as fast as possible and at a self-selected pace, respectively, without any external load. Thus, we assume that the STS test score between two different posture conditions, when performed at a self-selected pace (including maximal-effort speed), may be variable between individuals depending on the ability to keep balance and/or mobility. For this reason, the STS test score with the upright condition, which would minimize the effect of such ability as well as the trunk raising movement, may have been chosen as an independent variable to predict QF ACSA.

No significant correlation between QF ACSA and the time of STS test score was observed (Fig. 3). This is in agreement with a previous report [12] which showed that time of STS test score was not significantly correlated to the QF ACSA in 57 middle-aged and elderly population. The power of the STS test score was greater in males than in females, but time of STS test scores of females were significantly shorter than those of males (Fig. 2). We consider therefore that the time of STS test score by itself is not enough to evaluate the QF ACSA in the middle-aged and elderly population.

In terms of health sciences or clinical applications, the present results could help improve the determination of QF muscle size in the middle-aged and elderly population. Although atrophy of the QF is induced by aging [1, 2], disuse [27, 28], and the immobilization by an operation [36], which has been quantified by the MR imaging as a gold standard, the MR is not easily accessible and not suitable for the large-sample measurements. To solve this problem, the QF muscle size has been assessed by a power index of the STS test score [12]. The predictive equation of the present study could develop the estimation of the QF muscle size, and suggest that the STS test score with the upright condition is better than that with the stooping condition to estimate the QF ACSA. Therefore, the present finding would contribute to establishing the efficient measurement procedure in the practical situation, determining the QF muscle size of the middle-aged and elderly population.

In conclusion, the present study indicates that the power of STS test score with trunk in the upright condition, sex, and age could adequately estimate the QF ACSA of the middle-aged and elderly population. The STS test has been extensively used as a measurement of physical function for the elderly, owing to high easiness and safeness of the measurement. Therefore, the estimated predicting equation found in the present study, in relation to the difference in the posture during the test, should be useful in clinical and practical settings for the health promotion of the elderly.

## Abbreviations

ACSA, anatomical cross-sectional area; MR, magnetic resonance; MVC: maximal voluntary contraction; QF, quadriceps femoris; STS, sit-to-stand.
